# Restoration of angiogenic capacity of diabetes-insulted mesenchymal stem cells by oxytocin

**DOI:** 10.1186/1471-2121-14-38

**Published:** 2013-09-11

**Authors:** Yong Sook Kim, Jin Sook Kwon, Moon Hwa Hong, Wan Seok Kang, Hye-yun Jeong, Hye-jin Kang, Myung Ho Jeong, Youngkeun Ahn

**Affiliations:** 1 Heart Research Center, Chonnam National University Hospital, 42 Jebong-Ro, Dong-Gu, Gwangju 501-757, South Korea; 2 Research Laboratory of Cardiovascular Regeneration, Chonnam National University Hospital, 42 Jebong-Ro, Dong-Gu, Gwangju 501-757, South Korea; 3 Department of Cardiology, Chonnam National University Hospital, 42 Jebong-Ro, Dong-Gu, Gwangju 501-757, South Korea

**Keywords:** Diabetes, Angiogenesis, Stem cells, Oxytocin, Krüppel-like factor 2

## Abstract

**Background:**

Angiogenesis is the main therapeutic mechanism of cell therapy for cardiovascular diseases, but diabetes is reported to reduce the function and number of progenitor cells. Therefore, we studied the effect of streptozotocin-induced diabetes on the bone marrow-mesenchymal stem cell (MSC) function, and examined whether diabetes-impaired MSC could be rescued by pretreatment with oxytocin.

**Results:**

MSCs were isolated and cultured from diabetic (DM) or non-diabetic (non-DM) rat, and proliferation rate was compared. DM-MSC was pretreated with oxytocin and compared with non-DM-MSC. Angiogenic capacity was estimated by tube formation and Matrigel plug assay, and therapeutic efficacy was studied in rat myocardial infarction (MI) model.

The proliferation and angiogenic activity of DM-MSC were severely impaired but significantly improved by pretreatment with oxytocin. Krüppel-like factor 2 (KLF2), a critical angiogenic factor, was dramatically reduced in DM-MSC and significantly restored by oxytocin. In the Matrigel plug assay, vessel formation of DM-BMSCs was attenuated but was recovered by oxytocin. In rat MI model, DM-MSC injection did not ameliorate cardiac injury, whereas oxytocin-pretreated DM-MSC improved cardiac function and reduced fibrosis.

**Conclusions:**

Our results show that diabetes influenced MSC by reducing angiogenic capacity and therapeutic potential. We demonstrate the striking effect of oxytocin on stem cell dysfunction and suggest the use of oxytocin as a priming reagent in autologous stem cell therapy.

## Background

Cell therapy with autologous bone marrow-mesenchymal stem cells (MSC) is a promising and safe modality with the potential for vascular regeneration in the treatment of ischemic diseases. MSC can differentiate into vascular lineage cells and can be directly incorporated into newly formed vessels [1]. However, the initial clinical trials of stem cell therapy after myocardial infarction failed to reproduce the substantial benefits demonstrated in the preclinical animal studies, especially in elderly patients [2-5]. One possible reason for the conflicting results in cell therapy research is that the stem cells used in most of the animal studies were derived from young or healthy animals. Diabetes, obesity, or aging influence stem cell numbers and activities [6-11], thus the animal studies could not predict the outcomes of autologous stem cell therapy for a patient with diabetes or other risk factors [12,13].

Diabetes is widely recognized to be an independent risk factor for coronary heart disease, stroke, peripheral arterial disease, cardiomyopathy, and congestive heart failure [14-17]. Furthermore, diabetes is closely associated with poor neovascularization after ischemia [2]. Cells isolated from diabetic patients are significantly impaired in their ability to recover blood flow after ischemia compared with cells isolated from healthy donors [3]. In addition, diabetic rats fail to induce neovascularization for recovery from hind limb ischemia, partly as the result of a dysfunction of endothelial progenitor cells [6]. There is no doubt that diabetes is not the only factor associated with dysfunction of progenitor cells, but diabetes leads to significant cellular dysfunction such as poor migration, reduced proliferation, and poor vascular network formation [7]. Diabetes-related changes in stem cells or progenitor cells may not only account for the reduced proliferation rate of these cells but also for their limited angiogenic potential [18].

Oxytocin is a neurohypophyseal hormone expressed in the hypothalamus. Previous studies revealed that oxytocin induces cardiomyogenesis of embryonic stem cells [19] and adult Sca-1 (+) stem cells [20], stimulates the migration of endothelial cells [21], and increases the engraftment [22] and cardiac differentiation potency [23] of umbilical cord blood-derived mesenchymal stem cells in infarcted myocardium.

Krüppel-like factor 2 (KLF2), which was first cloned by Lingrel and colleagues [24], is emerging as a master regulator of endothelial quiescence, anti-inflammatory and antithrombotic properties, and vascular tone by activating atheroprotective and inhibiting atherogenic transcription [25]. Proangiogenic cells isolated from aged mice showed a lower level of KLF2 than do cells from young mice [26]. Despite growing evidence of cellular dysfunction, however, the signaling pathways of stem cells in various physiological and pathological niches remain to be investigated.

The potential disadvantage of autologous stem cells is that patients with diabetes may have a decline in number and regenerative capacity of bone marrow and circulating stem cells; thus, patients who have diabetes may have not only damaged myocardium but also a lessened capacity for repair.

In this study, we aim to compare bone marrow-MSCs obtained from non-diabetic and diabetic rats, and examine whether diabetes-insulted MSC could be rescued by pretreatment with oxytocin.

## Results

### Proliferation is reduced in DM-MSC and recovered by oxytocin

**Figure 1:**
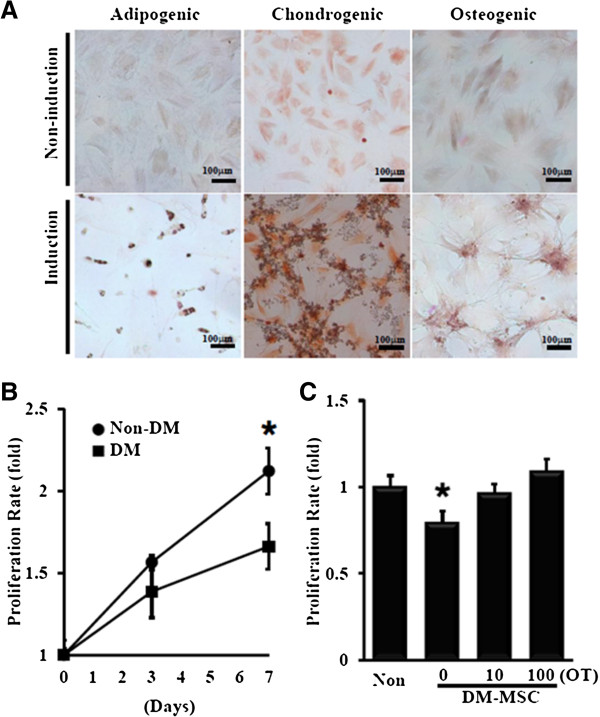
**Impaired proliferation of MSC from DM bone marrow was restored by oxytocin. (A)** Differentiation of MSC isolated from bone marrow was induced into adipogenic, chondrogenic, or osteogenic lineage. **(B)** The proliferation rate was significantly reduced in DM-MSC (n=5). **(C)** DM-MSC pretreated with oxytocin for 24 hours restored proliferation activity (n=5). * *P*<0.01 vs. Non-DM-MSC.

Hyperglycemia was induced 4 weeks after streptozotocin injection (Table 1), and the mortality was 29.17% in the diabetic group. Fasting blood glucose was substantially increased to 571.58 ± 57.64 g/dL. Isolated MSC were tested for their capabilities to differentiate into adipocytes, chondrocytes and osteoblasts *in vitro*. The results showed that MSC were capable of differentiating into adipocytes, chondrocytes and osteoblasts. The successful differentiations into adipocytes, chondrocytes and osteoblasts were identified by positive staining with Oil Red O, Alcian Blue-PAS and Alizarin Red S respectively (Figure 1A). Bone marrow-MSCs were isolated from non-DM rats (non-DM-MSC) and DM rats (DM-MSC) to perform the experiments. The proliferation rate of non-DM-MSC was lower to 79.43% than that of DM-MSC at day 7 (Figure 1B) and recovered by oxytocin treatment (Figure 1C).

**Table 1:** Animal characteristics after induction of diabetes

	**Non-diabetes**	**Diabetes**
***Day 1***	n = 10	n = 24
BW (g)	442.50 ± 20.43	461.47 ± 41.37
BG (mg/dL)	94.83 ± 8.70	92.47 ± 6.47
***Day 28***	n = 10	n = 17
BW (g)	558.33 ± 22.51	349.00 ± 67.96*
BG (mg/dL)	118.50 ± 9.27	571.58 ± 57.64*
HW (mg)	1.47 ± 0.01	1.16 ± 0.21*
TL (mm)	45.49 ± 0.78	44.60 ± 1.67
HW (g)/TL (mm)	32.28 ± 0.35	25.99 ± 4.17*

Data are presented as mean ± SD.

HW, heart weight; BW, body weight; BG, blood glucose; TL, tibia length.

*p < 0.01 vs non-diabetes.

### Tube formation and KLF2 are reduced in DM-MSC and recovered by oxytocin

**Figure 2:**
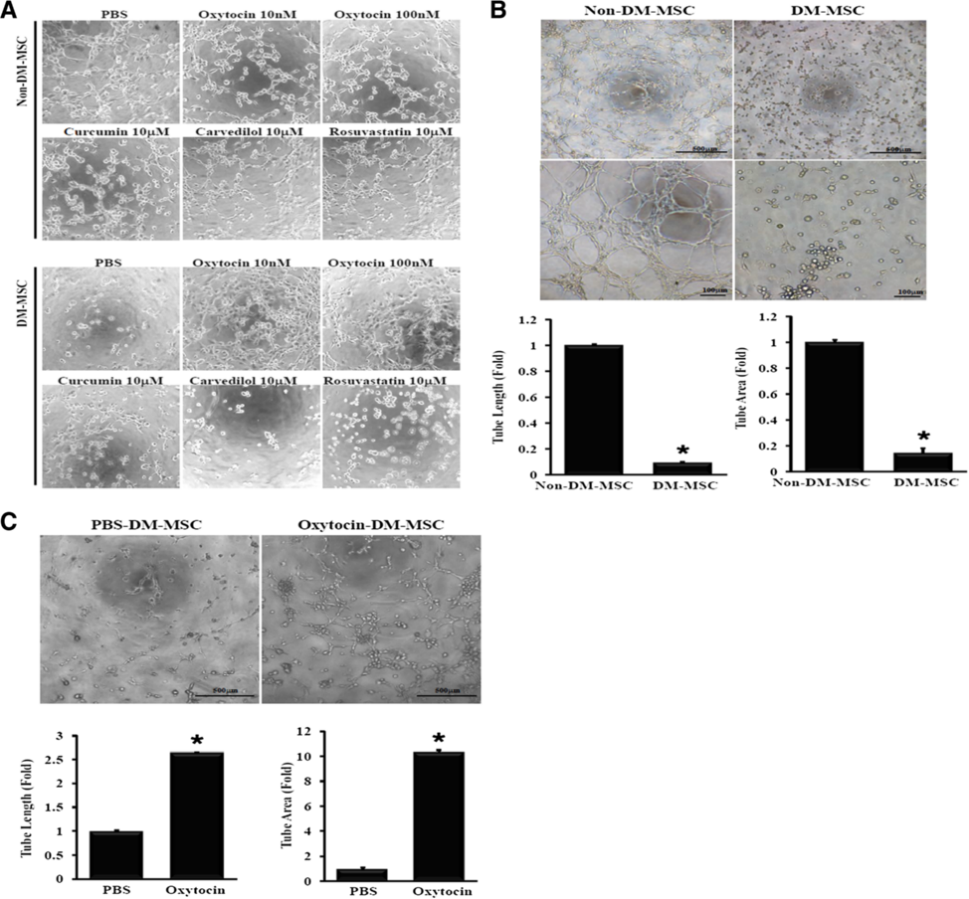
**Impaired tube formation of MSC from DM bone marrow was restored by oxytocin. (A)** MSCs were pretreated with PBS, oxytocin, curcumin, carvedilol, or rosuvastatin prior to tube formation assay. **(B)** Representative images from the *in vitro* angiogenesis assay showed the retarded tube formation in DM-MSC. Both tube length and tube area were measured and expressed in graphs (n=5). **(C)** Representative images of tube formation of DM-MSC after treatment with PBS or 100 nM of oxytocin for 24 hours were presented (n=4). * *p* < 0.01 vs. Non-DM-MSC.

**Figure 3:**
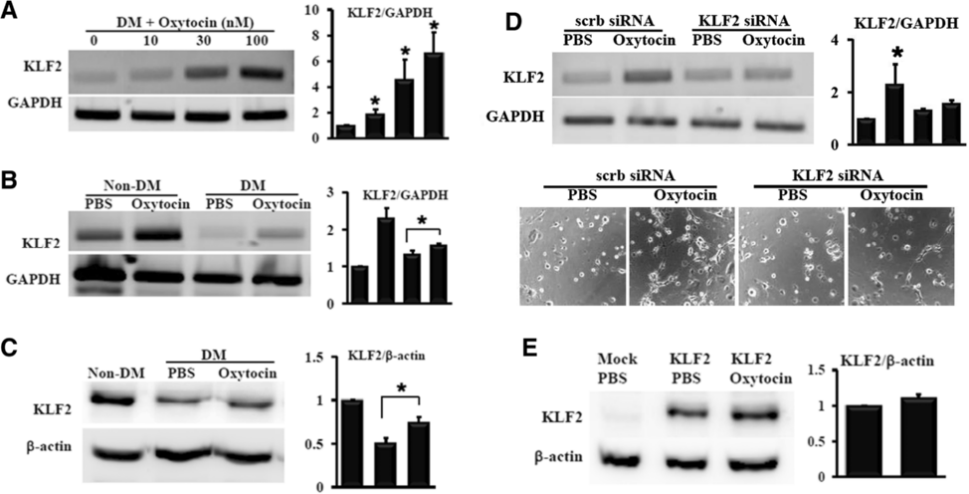
**Reduced KLF2 level of MSC from DM bone marrow was restored by oxytocin. (A)** KLF2 mRNA was assessed in DM-MSC treated with various concentrations oxytocin (n=3). **(B)** KLF2 mRNA was analyzed in MSC from non-DM or DM bone marrow treated with PBS or oxytocin for 24 hours (n=3). **(C)** The level of KLF2 protein was reduced in DM-MSC, whereas restored by oxytocin (n=3). **(D)** Oxytocin-induced KLF2 mRNA was assessed in DM-MSC transfected with scrambled siRNA or KLF2 siRNA (n=3). **(E)** After transfection with KLF2 plasmid for 24 hours, 293T cells were treated with PBS or oxytocin for 24 hours. KLF2 protein level was examined by Western blot (n=3). Quantification analysis was performed and data in the graph represented the mean ± standard deviation.

Angiogenic capacity of MSC was assessed by *in vitro* angiogenesis assay after treatment with PBS, oxytocin, curcumin, carvedilol, or rosuvastatin. Our previous reports demonstrated that oxytocin enhanced therapeutic activity of MSC [23], and curcumin blocked inflammatory responses [27]. Carvedilol, a drug for the treatment of hypertension and heart failure, inhibited endothelial inflammation. Rosuvastatin is widely prescribed to control hypercholesterolemia, and inhibited pro-inflammatory transcription factor nuclear factor-κB (NF-κB) [28]. As shown in Figure 1A, Non-DM-MSC was not affected by these reagents. DM-MSC treated with PBS, curcumin (10 μM), carvedilol (10 μM), and rosuvastatin(10 μM) showed retarded tube formation. On the other hand, tube formation was observed in oxytocin-treated DM-MSC. Non-DM-MSC showed significant tube formation on Matrigel within 3 hours, while few tubes were formed in DM-MSC. Tube length and tube area were lower in DM-MSC than in non-DM-MSC (0.10-fold and 0.15-fold of non-DM-MSC, respectively, Figure 2A). After pretreatment with 100 nM of oxytocin for 24 hours, tube length was higher by 2.64-fold and tube network area was higher by 10.34-fold in OT-DM-MSC than in PBS-treated DM-MSC (Figure 2B). To examine the relationship of KLF2 with the angiogenic capacity of MSC, KLF2 expression was analyzed. KLF2 mRNA was induced by oxytocin in dose-dependent manner in DM-MSC (Figure 3A). Both KLF2 mRNA and protein were reduced in DM-MSC and successfully induced by oxytocin pretreatment (Figure 3B,C).

To confirm whether the KLF2 is a mediator of oxytocin in DM-MSC, knockdown of KLF2 was induced by siRNA transfection. Oxytocin failed to induce both KLF2 mRNA and tube formation in KLF2 siRNA-transfected DM-MSC (Figure 3D). This result suggested that oxytocin might act on angiogenic potential of DM-MSC through KLF2 induction.

To examine whether KLF2 induction by oxytocin is specific to MSC, KLF2 plasmid DNA was transfected to 293T cells. Oxytocin treatment did not show any significant induction of KLF2 protein in 293T cells (Figure 3E).

### Impaired angiogenic capacity of DM-MSC is restored by oxytocin

**Figure 4:**
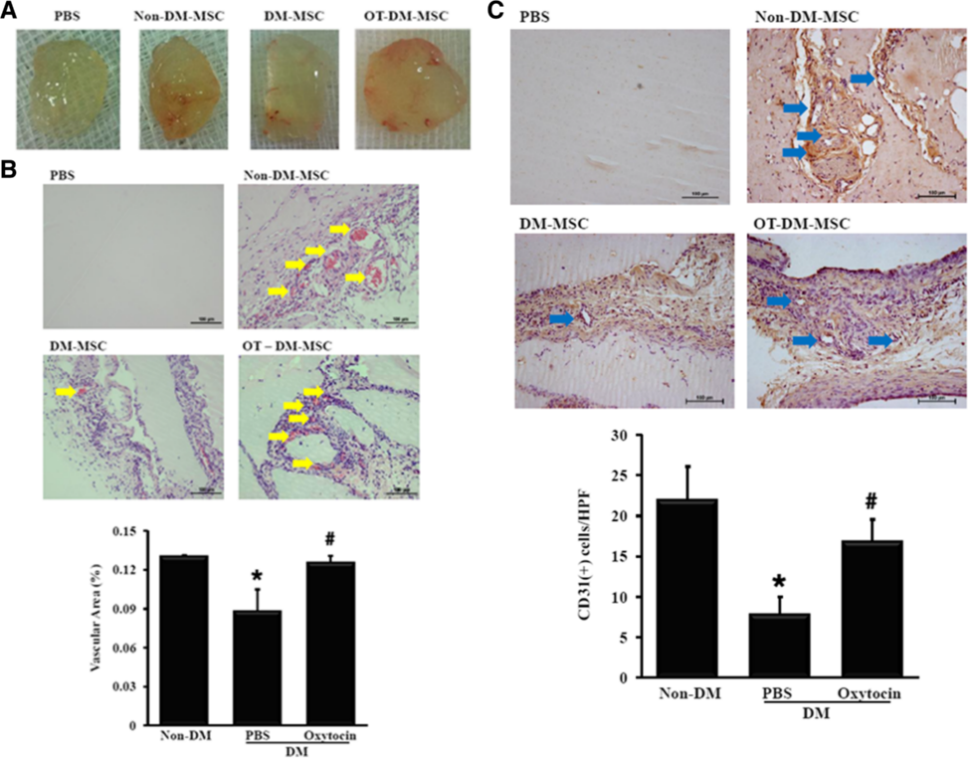
**Oxytocin significantly recovered neovascularization in the Matrigel plug assay (n=3). (A)** Gross images of Matrigel plugs harvested 2 weeks after subcutaneous injection into athymic nude mice. Note the substantial increased neovascularization in Matrigel containing DM-MSC compared with PBS-containing Matrigel. **(B)** Representative H&E-stained images of Matrigel plugs; arrows indicate vascular structures containing red blood cells. **(C)** The neovasculature formed by MSC in Matrigel plugs was quantified by immunohistochemical staining with anti-human CD31 IgG. *p< 0.05 vs. Non-DM-MSC; # p< 0.05 vs. OT-DM-MSC.

Gross findings were that neovascularization was induced in Matrigel plugs containing non-DM-MSC and OT-DM-MSC in the murine host (Figure 4A). Functional vessel formation was determined by measuring the red blood cell-containing area of vascular structures. The vascularized area was reduced to 0.68-fold that of plugs containing non-DM-MSC; by contrast, the vascularized area was improved 1.42-fold by oxytocin treatment compared with PBS treatment (Figure 4B). The vascularized area of DM-MSC was restored to a level similar to that of non-DM-MSC by oxytocin treatment (0.130 ± 0.01% in non-DM-MSC vs. 0.126 ± 0.01% in OT-DM-MSC, p > 0.05). The numbers of CD31-positive cells were significantly reduced by 0.36-fold in the DM-MSC-injected group compared with the non-DM-MSC group and were restored to 0.77-fold that of the non-DM-MSC group by oxytocin treatment (Figure 4C).

### Introducing exogenous KLF2 DNA into DM-MSC contributes to restoration of angiogenic activity of DM-MSC

**Figure 5:**
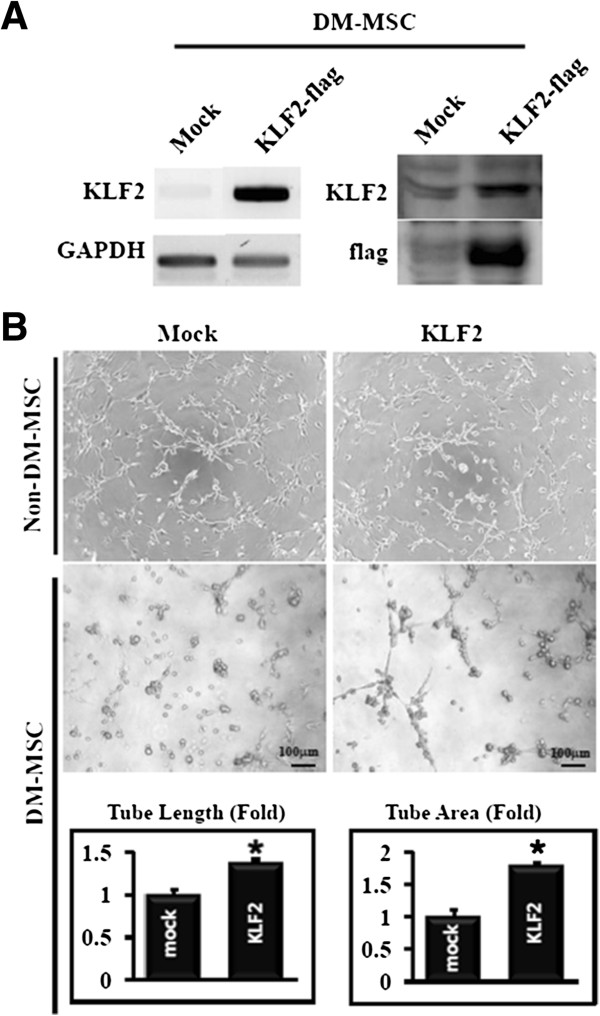
**Enforced expression of KLF2 into DM-MSC recovered angiogenic activity (n=3). (A)** Transfected KLF2 was measured by RT-PCR and Western blot against KLF2 and flag. **(B)** After transfection to non-DM-MSC or DM-MSC, tube formation was assessed and analyzed. *p < 0.05 vs mock-transfected DM-MSC.

To understand whether the loss of angiogenic potential is associated with down-regulated KLF2 in DM-MSC, KLF2 plasmid DNA was transfected into DM-MSC before the angiogenesis assay. After DNA transfection, KLF2 mRNA and KLF2 protein were significantly induced at 1 day and 2 days, respectively. In addition, the expression of flag, a tagging protein of transfected KLF2, was also well shown in KLF2-transfected MSC (Figure 5A). Non-DM-MSC showed no changes in tube formation after DNA transfection. Tube length was increased 1.37-fold and tube area was increased 1.79-fold in KLF2-overexpressing DM-MSC compared with that of DM-MSC transfected with the empty vector gWIZ (Figure 5B). These data showed that the upregulation of KLF2 by oxytocin in DM-MSC was consistent with the enhanced blood perfusion and capillary density in the ischemic limbs that received OT-DM-MSC.

### Restored therapeutic effects in myocardial infarction (MI) by OT-DM-MSC

**Figure 6:**
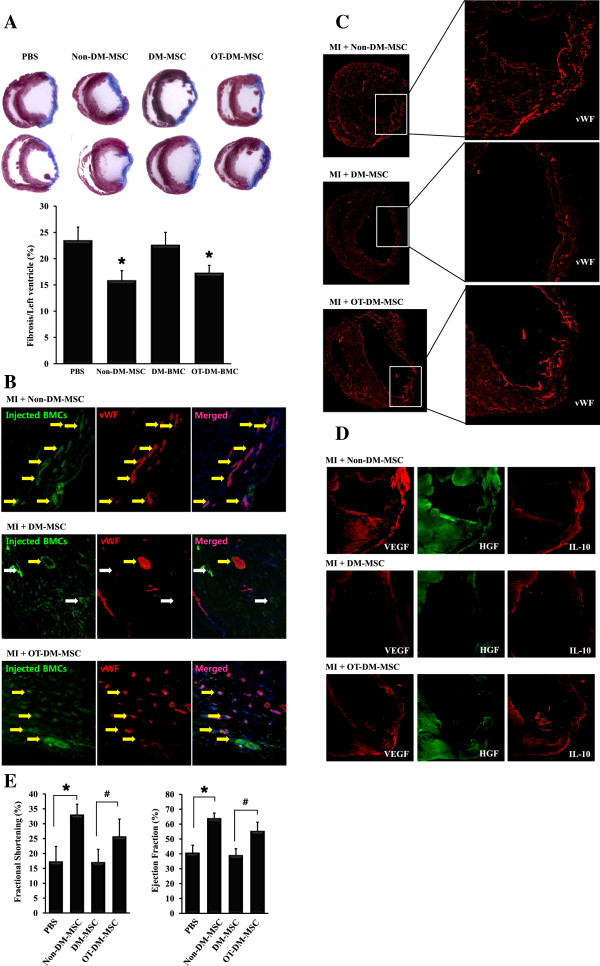
**Cardiac fibrosis, expressions of angiogenic factors, and cardiac function were ameliorated by injection of OT-DM-MSC in infarcted myocardium. (A)** Representative images showed fibrosis was significantly reduced in Non-DM-MSC and DM-MSC-injected heart. **(B)** Representative images of GFP-labeled MSCs (green) injected in infarcted myocardium showed expression of von Willebrand factor (vWF, red). The merged images showed vWF-expressing MSC (yellow arrows) and none-expressing MSC (white arrows). **(C)** Distribution and degree of vWF expression in the whole heart. Less vWF and restored vWF were observed in DM-MSC injected heart and DM-MSC injected heart after MI, respectively. **(D)** Expressions of vascular endothelial growth factor (VEGF), hepatocyte growth factor (HGF), and interleukin-10 (IL-10) in infarcted myocardium were examined. **(E)** Echocardiographic evaluations were performed 2 weeks after cell injection. * *p* < 0.01 vs PBS; # *p* < 0.01 vs DM-MSC.

The therapeutic effect of DM-MSC was examined in MI model. MSCs were injected into infarcted myocardium after 7 days after MI. Histology and cardiac function were assessed 14 days later. Compared with that in the PBS-injected heart, cardiac fibrosis was reduced to 67.65% in non-DM-MSC injected (*p*<0.01), 96.25% in DM-MSC injected (*p*>0.01), and 73.68% in DM-MSC injected (*p*<0.01) hearts (Figure 6A). GFP-labeled MSCs (green) stained with vWF (red) were frequent in non-DM-MSC or DM-MSC injected hearts, whereas seldom observed in DM-MSC injected heart (Figure 6B). To examine the angiogenesis in myocardium after MI, vWF expression in the whole heart was imaged. More vWF expression was observed in non-MD-MSC or OT-DM-MSC injected heart, whereas less was in DM-MSC injected heart (Figure 6C). Cytokines such as vascular endothelial growth factor (VEGF), hepatocyte growth factor (HGF), and interleukin-10 (IL-10) were also examined (Figure 6D). They were induced in Non-DM-MS-injected myocardium, whereas hardly expressed in DM-MSC-injected myocardium. In OT-DM-MSC-injected heart, VEGF, HGF, and IL-10 were significantly induced compared with in DM-MSC-injected heart. To verify the cardiac function, fractional shortening (FS) and ejection fraction (EF) were assessed. At 2 weeks, FS and EF were remained unchanged in DM-MSC injected group compared with PBS injected group. On the other hand, cardiac function was recovered in non-DM-MSC injected group and DM-MSC injected groups (Figure 6D).

## Discussion

MSC are one of the most actively studied stem cell sources for treating various cardiovascular diseases. Autologous MSC application is clinically available without ethical issues or immunological problems. The use of autologous MSC, however, is affected by factors such as aging or systemic diseases, which may contribute to the functional impairment of stem cells. Diabetes is one of the risk factors for cardiovascular diseases, and type 1 diabetes is a disorder characterized by hyperglycemia and a proinflammatory state [29,30]. Our results showed that diabetes impairs the neovascularization of bone marrow-derived MSC, and these findings are consistent with previous reports [31,32]. Several reports demonstrated diabetes exerted a detrimental effect on stem cells or progenitor cells. Endothelial progenitor cells obtained from type 1 diabetes patients [31] or streptozotocin-induced diabetes mice [33] showed the significant reduction of circulating cell number and cellular function. Prolonged exposure to high glucose condition has drastic effects on the differentiation potential, proliferation capacity, and cell survival of adipose tissue-derived MSC [9]. To overcome, we searched for a priming reagent to restore the angiogenesis activity of DM-MSC, and we found a transient treatment of DM-MSC with oxytocin for 24 hours improved tube formation capacity.

Oxytocin, a neurohypophyseal nonapeptide, modulates social recognition, emotion, and the female reproductive system [34]. In previous studies, we provided evidence for beneficial roles of oxytocin in umbilical cord blood-derived mesenchymal stem cells [22,23].

We next explored which effecter was controlled by oxytocin to restore the angiogenic potential. A previous report showed that cell number is reduced and KLF2 expression is repressed in proangiogenic cells as a result of senescence [26]. KLF2 is a well-known zinc-finger transcriptional regulator that is involved in endothelial development, functional regulation, and angiogenesis [25,35,36]. On the other hand, KLF2 inhibited function and expression of hypoxia-inducible factor α (HIF1-α) in hypoxia-mediated endothelia angiogenesis [37]. This discrepancy might be resulted from different inducers of angiogenesis. KLF2 might be required for development of physiological or therapeutic angiogenesis, and regulation of inflammation or hypoxia-induced angiogenesis. In this study, we focused on intrinsic angiogenic potential of MSC rather than on pathological lesion. We found that KLF2 was highly expressed in normal MSC, whereas it was significantly repressed in DM-MSC. Our data and those of a previous report [26] show that down-regulated KLF2 is closely associated with aggravation of the angiogenic potential of stem/progenitor cells. In addition to angiogenesis, oxytocin-treated DM-MSC successfully induced as well anti-inflammatory IL-10 as angiogenic cytokines such as VEGF and HGF in injected infarct lesion.

The most important limitation in this study is that we did not address the exact mechanism of KLF2 induction by oxytocin.

Taken together, these results show that DM-MSC showed impaired angiogenesis and reduced KLF2, which were restored by oxytocin treatment. Stem cell therapy with autologous MSC in persons with risk factors such as diabetes, aging, or systemic disorders is expected to be less effective than stem cell therapy in the preclinical animal experiments. For therapeutic application, therefore, the future challenge is to establish a safe procedure for patients with risk factors to normalize the function of endogenous stem cells before autologous stem cell application. We suggest that strong potential exists for translating the results of the present study to human trials, which would be very beneficial to patients who are at risk of cardiovascular disease in the setting of diabetes.

## Conclusions

In the present study, we evaluated a putative relation between diabetes and poor angiogenesis of stem cells in the myocardial infarction model. Our data showed that the cellular function of MSC might be disturbed by exposure to the diabetic niche. In addition, we studied oxytocin and KLF2 in relation to the restoration of the angiogenic potential of DM-MSC.

## Methods

### Experimental hyperglycemia

This study was approved by the Chonnam National University Institutional Animal Care and Use Committee (CNU AICUC-H-2010-11). Twelve-week-old adult male Sprague–Dawley rats (Jung Ang, Korea) received intraperitoneal injections of streptozotocin (65 mg/kg, Sigma, USA) dissolved in 50 mM sodium citrate buffer (pH 4.5) to induce type 1 diabetes. Age-matched controls were injected with an equivalent volume of saline. Fasting blood glucose levels were measured in tail veins, and rats with a blood glucose level > 250 mg/dl were considered to be diabetic and were included in the study.

### Culture of MSC and 293T cells

MSCs were isolated and cultured as described in previous reports [38-40]. MSCs were obtained from the tibia and femur under sterile conditions by using a syringe to flush the cavity out with warmed phosphate-buffered saline (PBS), collected by centrifugation, and resuspended with DMEM with 10% fetal bovine serum. Cells were plated into culture dishes, and nonadherent cells were removed by changing the medium after 72 hours. All cells used in this study were from the third and fourth passages of MSC. To identify stemness, adipogenic, chondrogenic, and osteogenic differentiations were performed in monolayer culture of MSC by using Stem Cell Differentiation Kits (Life technologies, USA). Briefly, MSC were cultured in growth medium or differentiation medium. After 7 days, cells were fixed in formalin and stained with Oil Red O (Sigm-Aldrich, USA), Safranin O (Sigm-Aldrich, USA) and Alizarin Red S (Sigm-Aldrich, USA) staining to determine the adipogenesis, chondrogenesis and osteogenesis, respectively.

Oxytocin (100 nM, Sigma-Aldrich, USA), curcumin (Sigma-Aldrich, USA), carvedilol (kindly provided by Chong Kun Dang Pharm., Korea), and rosuvastatin calcium (kindly provided by AstraZeneca Korea) were added to the growth medium for 24 hours for the experiments. 293T cells were purchased from the ATCC (USA) and maintained in Dulbecco’s Modified Eagle’s Medium (Invitrogen, USA) supplemented with 10% fetal bovine serum (Invitrogen, USA).

### Proliferation assay of MSCs

Cells were plated on 48-well plate and the proliferation rate was measured with WST-1 (Roche Applied Science, USA). Briefly, WST-1 reagent was added at each time points, and the absorbance was measured at 490nm after incubation for 2 hours.

### *In vitro* angiogenesis assay

Tube formation was assayed by using an in vitro angiogenesis assay kit (Chemicon, USA). Cells (1 × 10^4^) were plated onto matrix gel-coated 96-well plates and were cultured in DMEM without serum. Tube formation was monitored and photographed by using an inverted microscope (Olympus CRX41, Japan), and images were analyzed by using Image-Pro software (Media Cybernetics, Inc., USA). Angiogenic activity was quantified by measuring tube length and tube area. Total tube length in four fields per well was averaged, and three wells were used to produce one value per condition.

### Reverse transcriptase-polymerase chain reaction (RT-PCR)

To compare the mRNA expression level of KLF2, RT-PCR was done as previously described [23]. Primers were designed from Bioneer (Daejeon, Korea) and the sequences were as follows: human KLF2 forward, caagacctacaccaagagttcgca, reverse, tacatgtgccgtttcatgtgcagc; rat KLF2 forward, ttgcagctacaccaactgcg, reverse, tgtcgcttcatgtgcagagc; rat glyceraldehydes 3-phosphate dehydrogenase (GAPDH) forward, ggccaaggtcatccatga, reverse, tcagtgagcccaggatg. Band densities were estimated using the Scion image 4.02 software (Scion Corporation, USA).

### Western blot analysis

Cells were lysed and analyzed as previously described [23] with blotting with KLF2 antibody (Novus Biologicals, USA) and flag antibody(Sigma-Aldrich, USA). β-actin (Sigma, USA) was used as a loading control. Band densities were estimated using the Scion Image 4.02 software (Scion Corporation, USA).

### Plasmid DNA or siRNA transfection

KLF2 plasmid DNA was purchased from OriGene Technologies (Rockville, USA). MSC were transfected with KLF2 plasmid DNA or gWIZ mammalian expression vector (Genlantis, USA) by using Lipofectamine 2000 (Invitrogen, USA) according to the manufacturer’s instruction. Overexpression of KLF2 was confirmed by RT-PCR and Western blot at 1 day and 2 days after transfection, respectively. To study the effect of oxytocin on KLF2 expression, KLF2 plasmid DNA was transfected to 293T cells for 1 day, and then cells were treated with oxytocin or PBS for 1 day. To knock down KLF2, KLF2 siRNA and scrambled siRNA (Bioneer, Korea) were transfected to DM-MSC with Lipofectamine 2000. After 2 days, siRNA transfected cells were treated with oxytocin (100 nM) for 24 hours.

### Matrigel plug assay

PBS or 2 × 10^5^ cells were mixed with phenol-red-free Matrigel (Matrigel™ Basement Membrane Matrix High Concentration, BD Biosciences, USA), and subcutaneously injected into 7-week-old male Balb/c athymic nude mice. After 2 weeks, the Matrigel plugs were harvested and processed for analysis. To estimate the degree of vascularization, H&E-stained digital images were analyzed by measuring the erythrocyte-filled area and expressing that as a percentage of the total area of Matrigel.

### Rat model of myocardial infarction (MI)

Male Sprague–Dawley rats (weighing 200–230 g, Jung Ang Animals, Korea) were used to induced MI previously described [23]. After 7 days, rats were randomly divided into 4 groups (n=5 each group) and anesthetized for reoperation. To visualize the injected MSCs for immunohistochemical examination, cells were infected with lenti-GFP virus for 48 hours and washed out with PBS before injection. DM-MSCs were pretreated with PBS or oxytocin (100 nM) for 24 hours before injection. Rats were injected with PBS alone, non-DM-MSC, DM-MSC, or oxytocin-pretreated DM-MSCs (OT-DM-MSC) into the peri-infarct area. MSCs (5 × 10^5^ diluted in 100 μL of PBS) were directly injected into the peri-infarct area. Finally, the heart was repositioned in the chest, and the chest was closed. The animals remained in a supervised setting until they were fully conscious.

### Echocardiography

After 2 weeks, echocardiography was performed as previously described [23].

### Immunohistochemical staining

For immunohistochemical analysis, slides were stained as previously described [23] with primary antibodies against von Willebrand factor (vWF, Biomedicals, Switzerland, 1:100), GFP (Abcam, USA), VEGF (Santa Cruz, USA), HGF (Santa Cruz, USA), and IL-10 (Abcam, USA).

### Statistical analysis

All data are presented as means ± SDs. P values were calculated by using the unpaired Student’s t-test. For analysis of the in vivo ischemia experiments, the Scheffe's test was performed for multiple comparisons after ANOVA between the groups. A P < 0.05 was considered statistically significant.

## Abbreviations

MSC: Mesenchymal stem cell; BM: Bone marrow; DM: Diabetes mellitus; MI: Myocardial infarction; KLF2K: Rüppel-like factor 2.

## Competing interests

The authors declare that they have no competing interests.

## Authors’ contributions

YSK designed this study, performed experiments in cell culture, Mtrigel plug assay, myocardial infarction surgery, transfection, and drafted manuscript. JSK performed myocardial infarction surgery and cared experimental rats. MHH performed cell isolation, RT-PCR, Western blot, and tube formation assay. WSK performed plasmid manipulation and induction of diabetes in rats. HJ and HK performed histologic studies. MHJ contributed to the conception of this study. YA contributed the conception and design of this study. All authors read and approved the manuscript.
